# Familial Predisposition to Anterior Cruciate Ligament Injury: A Systematic Review with Meta-analysis

**DOI:** 10.1007/s40279-022-01711-1

**Published:** 2022-07-12

**Authors:** Sara Hasani, Julian A. Feller, Kate E. Webster

**Affiliations:** 1 La Trobe University, Melbourne, VIC Australia; 2 OrthoSport Victoria, Epworth HealthCare, Melbourne, VIC Australia

## Abstract

**Background:**

Having a family history of anterior cruciate ligament (ACL) injury has been investigated in the literature but few studies have focused on this factor specifically or reported their outcomes by sex.

**Objective:**

We aimed to systematically review family history as a risk factor for sustaining a primary ACL injury and the impact it has on ACL graft rupture or contralateral ACL injury in male and female individuals.

**Methods:**

A literature search was completed in seven databases from inception until March 2021 to investigate primary and subsequent ACL injuries in those with a family history of ACL injury. Articles were screened by prespecified inclusion criteria, and the methodological quality of each study was determined. Study results were combined using an odds ratio (OR) meta-analysis. Subgroup analysis was also completed by sex for primary ACL injury, as well as by graft rupture and contralateral ACL injury for subsequent ACL injuries.

**Results:**

Twelve studies were acquired for systematic review and meta-analysis. Four studies that investigated primary ACL injury, seven that investigated ACL graft and/or contralateral ACL ruptures and one study that investigated both primary and subsequent ACL injury. Having a family history of ACL injury increased the odds of injury across all outcomes. Those with a family history had a 2.5 times greater odds for sustaining a primary ACL injury (OR 2.53 [95% confidence interval [CI] 1.96–3.28, *p* < 0.001)]. There was no significant difference of injury odds for primary ACL injury when analysed by sex. Family history of ACL injury was found to increase the odds of subsequent ACL injury by 2.38 (95% CI 1.64–3.46, *p* < 0.001) and was significant for both graft ruptures (OR 1.80 [95% CI 1.20–2.71, *p* = 0.005]) and contralateral ACL injuries (OR 2.28 [95% CI 1.28–4.04, *p* = 0.005]). When compared directly, the odds of sustaining a graft rupture versus a contralateral ACL injury were similar for those with a family history. Outcomes were not frequently reported by sex for subsequent ACL injuries.

**Conclusions:**

Having a family history of ACL injury more than doubles the odds of sustaining a primary or subsequent ACL injury. However, if a family history of ACL injury is present, the sex of the athlete does not increase the risk for primary injury nor is there a difference in the risk for a subsequent graft rupture compared to a contralateral ACL injury.

**Clinical Trial Registration:**

PROSPERO: CRD42020186472.

## Key Points

**Table:** 

Having a family history of an anterior cruciate ligament (ACL) injury increases the odds of sustaining a primary ACL compared with those without a family history by 2.5 times.
Female and male individuals with a family history are at the same increased odds for primary ACL injury.
The risk of sustaining a subsequent ACL injury are increased by 2.4 odds for those with a family history of ACL injury.
This increased risk is the same for ACL graft ruptures and contralateral ACL injuries.

## Introduction

An anterior cruciate ligament (ACL) rupture is a devastating knee injury that most commonly occurs whilst playing sport. Seventy percent of ACL injuries occur from non-contact athletic movements such as side-cutting, pivoting or landing [1, 2]. A significant amount of research has been undertaken to investigate why an ACL injury may occur at a specific moment in time during such common movements to try to identify risk factors and potential causes for injury. The aetiology has been found to be complex and there are multiple intrinsic and extrinsic factors that have been investigated and identified in the literature [2–4].

Having a family history of ACL injury has been investigated as a common risk factor for ACL injury. However, the definition of “family history” has been variable amongst studies with some reporting on parents [5, 6] or siblings alone [7] and another reporting beyond first-degree relatives [8]. Despite this, having a family history of ACL injury and being female have both been reported to increase the risk of a primary ACL injury by two to three times [4, 9–11]. Myer et al. [12] found that male participants with a primary ACL injury had a higher prevalence of family history when compared with female participants. However, most studies fail to report the outcomes of those with a family history by sex. As a result, the impact of these two factors together has not been thoroughly investigated and warrants further examination.

Following an ACL injury, an athlete will most commonly proceed with surgical reconstruction of the ACL. Despite what is usually an extensive rehabilitation process [13], there is an increased risk of reinjury if that athlete returns to sport [14]. The rate of graft ruptures and subsequent disruption of the contralateral ACL (CACL) have been found to be relatively consistent in the literature at 10–12% at 3–5 years after surgery [14–17]. The outcomes of those with a family history and their risk for sustaining a subsequent ACL injury have been more commonly reported in the literature than for those of primary ACL injuries. Some studies have found the rate of subsequent ACL injuries to the ACL graft or the CACL to be greater in those with a family history [18, 19] whilst another was inconclusive [15].

The first aim of this review was to perform a meta-analysis of the available studies to determine if family history is a risk factor for primary ACL injury. The second aim was to determine if those with a family history of ACL injury are at greater risk of sustaining a subsequent ACL graft rupture or CACL injury compared to those without. Our final aim was to investigate if the odds for ACL injury are the same for male and female individuals who have a family history.

## Methods

This systematic review with meta-analysis was conducted according to the Preferred Reporting Items for Systematic Reviews and Meta-Analyses (PRISMA) guidelines [20]. The study protocol was pre-registered with PROSPERO (registration: CRD42020186472).

The PICO (population, intervention, comparator, and outcomes) concept was used to formulate our research question [21]. The population was those with an ACL injury in whom the family history was known, and the intervention or problem was primary or subsequent ACL injury following surgical reconstruction. The comparator and outcome were not used to further define the search as we wanted to keep our search broad to yield as many studies as possible. A literature search (Table 1) was completed with MEDLINE, Embase, AMED, CINAHL, SPORTDiscus, Web of Science and Cochrane library databases from inception until April 2020 and then updated March 2021. All studies were imported to a reference-manager software (EndNote X9 software, Clarivate, Philadelphia, PA, USA; Thomson Reuters) and duplicates removed. All titles and abstracts were screened and studies not relating to ACL injury were removed. Full texts were obtained for all remaining studies and subsequently reviewed by two authors (SH and KW) for any mention of familial history, hereditability or genetics. In addition, all references from the full-text articles were screened for additional studies.

**Table 1:** Search strategy

Database	Search terms	Results 1 and 2 combined
MEDLINE	1. Heredity/ OR Heredit* OR "Family History" OR Familial OR Genetic 2. AND "Anterior Cruciate Ligament/" OR "Anterior Cruciate Ligament Injury/" OR "Anterior Cruciate Ligament Reconstruction/" OR "Anterior Cruciate Ligament Rupture/" OR "Anterior Cruciate Ligament*" OR ACL	965
Embase	1. Heredity/ OR Heredit* OR "Family History" OR Familial OR Genetic 2. AND "Anterior Cruciate Ligament/" OR "Anterior Cruciate Ligament Injury/" OR "Anterior Cruciate Ligament Reconstruction/" OR "Anterior Cruciate Ligament Rupture/" OR "Anterior Cruciate Ligament*" OR ACL	769
AMED	1. Heredity/ OR Heredit* OR "Family History" OR Etiology/ Epidemiology/ Genetics / OR Familial OR Genetic 2. AND "Anterior Cruciate Ligament/" OR "Anterior Cruciate Ligament*" OR ACL	75
CINAHL	1. Heredity or genetics or family history 2. AND (anterior cruciate ligament or acl) OR anterior cruciate ligament injury OR (anterior cruciate ligament reconstruction or acl reconstruction or aclr)	309
SPORTDiscus	1. Heredity or genetics or family history 2. AND anterior cruciate ligament or acl or acl injury	306
Web of Science	1. Heredity OR Heredit* OR "Family History" OR Familial OR Genetic* 2. AND "Anterior Cruciate Ligament*" OR ACL OR ACLR	1056
Cochrane Library	1. Heredity/ OR Heredit* OR "Family History" OR Familial OR Genetic* 2. AND "Anterior Cruciate Ligament/" OR "Anterior Cruciate Ligament Reconstruction/" OR "Anterior Cruciate Ligament*" OR ACL OR ACLR	31
Total		3511

### Inclusion and Exclusion Criteria

For studies to be included, they needed to clearly report the outcomes for ACL injury in participants with and without a family history of ACL injury. Studies were excluded if they were on animals, purely genetic or genome studies with no mention of family history, bilateral ACL reconstructions, or if the study had a control group which the reviewers agreed was not comparable or exposed to the same risk factors, i.e. a sporting population compared to a non-athletic population or an ACL-injured population compared to another knee injury or condition. Both prospective and retrospective study designs were accepted for studies that investigated primary ACL injury as well as cohort and case–control studies. Studies that investigated subsequent ACL graft or CACL injury were also excluded if they used mainly allograft or synthetic grafts for ACL reconstruction or had less than 2 years of follow-up for graft rupture or CACL injury outcomes. No editorials, case studies, reviews or other systematic reviews were included. The quality of each study was reviewed with the National Heart Lung and Blood Institute Quality Assessment Tool [22]. Quality assessment ratings were established on the general guidelines published by the developers of the quality assessment tool. A score ≥ 9 was considered good, a score of 6–8 was considered fair and a score ≤ 5 was considered poor [23]. All conflicts were resolved by discussion between SH and KW and any disputes were reviewed by a third author, JF.

### Data Collection

All studies were reviewed for the following information: authors, journal name, year of publication, language, type of study, number of participants, age range and mean, total number of female and male individuals, author’s definition of family history, cohort groups, and information and surgery details if applicable. The studies were then divided into two groups: those relating to primary ACL injury (Table 2) and those relating to subsequent graft rupture or CACL injury (Table 3).

**Table 2:** Characteristics of the studies included in the primary ACL injury meta-analysis

Author (year)	Study design	Definition of family history	No. of participants (% family history)	% Female (% male)	Age range, years (mean)	Population description	Score (quality)
Flynn et al. (2005) [8]	Matched case–control retrospective	Investigated first-degree, second-degree and third-degree relatives	342 (17%)	56% (44%)	13–79 (23)	Case: patients with an ACL rupture at a private clinic over an unknown period including paediatric and adult cases Control: university teams with no major knee injury history	6/12 (fair)
Hägglund and Waldén (2016) [27]	Cohort prospective	All first-degree relatives	4301 (15%)	100% (0%)	12–17 (14.1)	Swedish female youth football (soccer) teams over 1 season	12/13 (good)
Vacek et al. (2016) [5]	Matched case–control prospective	Parent only	327 (14%)	67% (33%)	14–23 (not given)	Caseinjured athletic students from high schools and colleges located throughout the state of Vermont over 4 years Control: randomly selected team-mates of the injured control subject. Matched by age, sex and sport	9/12 (good)
Westin et al. (2016) [6]	Cohort retrospective	Parent only	418 (20%)	55% (45%)	High school (20.5)	Elite alpine skiers who studied at a Swedish alpine ski high school over 6 years	9/13 (good)
Bram et al. (2020) [31]	Case–control retrospective	All first-degree relatives	717 (20%)	53% (47%)	< 18 (case: 14.9, control 13.9)	Case: paediatric patients with an ACL rupture at a private clinic over 9 years Control: paediatric cases with uninjured knees from a concussion clinic	9/12 (good)

*ACL* anterior cruciate ligament

**Table 3:** Characteristics of the studies included in the subsequent ACL injury meta-analysis

Author (year)	Study design^a^	Definition of family history	No. of participants (% family history)	% Female (% male)	Age range, years (mean)	Follow-up period (mean)	Population description	Score (quality)
Bourke et al. (2012) [19]	Case series	All first-degree relatives	669 (23%)	36% (64%)	13–62 (29)	Data collected at 2, 5, 10 and 15 years. Follow-up data at 5 years are used in the meta-analysis	Patients with an ACL rupture at a private clinic over a 2-year period	9/9 (good)
Goshima et al. (2014) [29]	Case series	All first-degree relatives	233 (16%)	54% (46%)	14–52 (21)	Minimum 2 years	Patients with an ACL rupture at a private clinic over a 3-year period	7/9 (good)
Webster et al. (2014) [16]	Case series	All first-degree relatives	561 (42%)	34% (66%)	(28)	Minimum 3 years (4.8)	Patients with an ACL rupture at a private clinic over a 5-year period	11/12 (good)
Morgan et al. (2016) [26]	Case series	All first-degree relatives	242 (32%)	43% (57%)	13–18 (16)	Data collected at 2, 5, 10 and 15 years. Follow-up data at 5 years are used in the meta-analysis	Paediatric patients with an ACL rupture at a private clinic over a 6-year period	9/9 (good)
Lai et al. (2018) [30]	Case series	All first-degree relatives	101 (33%)	0% (100%)	(23.5)	2–16 years	Athletes with an ACL rupture in the Australian Football League	8/9 (good)
Pierce et al. (2018) [28]	Case series	All first-degree relatives	290 (2%)	42% (48%)	15–62 (24)	2–5 years (3 years)	Patients with an ACL rupture at a private clinic over a 3-year period	9/12 (good)
Bram et al. (2020) [31]	Cohort	All first-degree relatives	450 (25%)	53% (47%)	< 18 (14.9)	(4.3) ± 2.1 years	Paediatric patients with ACL rupture at a private clinic over 9 years	9/12 (good)
Mardani-Kivi et al. (2020) [7]	Cross-sectional	Siblings only	836 (19%)	21% (79%)	> 16 (34)	5–8 (6.5)	Patients with an ACL rupture from two private clinics over a 4-year period	8/9 (good)

*ACL* anterior cruciate ligament

^a^Please note that study design refers to the design as listed in the published abstract

All studies were analysed, and the raw data extracted for the number of participants with and without a family history into groups for ACL rupture, graft rupture or CACL rupture. The data were also separated by sex for the studies that investigated primary ACL injury. Authors of all five papers in the primary ACL injury group were contacted for additional information if the raw data were not able to be obtained from the published article. We are grateful that all the authors were able to provide the additional information. If there was any potential for the cohorts to be the same across multiple studies, the authors were contacted for clarification. Studies were excluded from the meta-analysis if 30% or more of the participants overlapped with another cohort.

### Data Analysis

A comprehensive meta-analysis was performed using Review Manager (RevMan) [Computer Program] Version 5.4, The Cochrane Collaboration, 2020. An odds ratio (OR) reporting the odds of sustaining an injury was calculated for those with a family history of ACL injury from the number of events and sample size with a 95% confidence interval (CI) using a random-effects model. For a primary ACL injury, this was also analysed by sex. For subsequent ACL injuries, this was separated by graft and contralateral ruptures.

## Results

### Literature Search

**Fig. 1:**
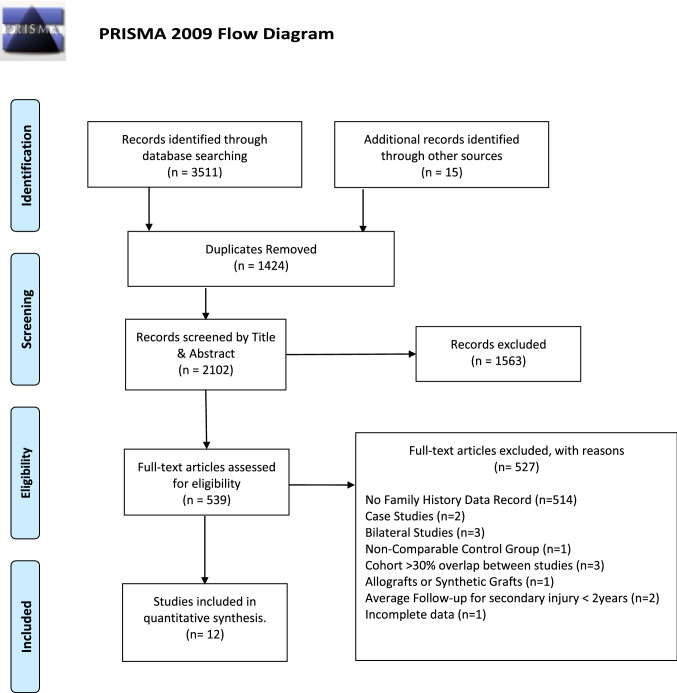
Preferred Reporting Items for Systematic Reviews and Meta-Analyses (PRISMA) flow diagram of the search strategy

A total of 3511 records were identified through database searches and 15 from other sources. Following the removal of duplicates, 2102 studies remained. These studies were screened by title and abstract and those clearly not related to the topic were removed. All 539 studies related to ACL injury were screened in full and checked against the inclusion and exclusion criteria. Three studies, Salmon et al., Thompson et al., and Salmon et al. [15, 24, 25], were from one large cohort that was included in Bourke et al. [19] and were excluded from the same meta-analysis. Twenty-seven percent of the participants included in Morgan et al. [26] were also from Bourke et al. [19]; however, this was below our 30% exclusion criterion and therefore Morgan et al. [26] was included in the same meta-analysis as Bourke et al. [19]. This process yielded a total of 12 studies for the systematic review and meta-analysis. A flowchart of this process is presented in Fig. 1.

From the 12 studies, we were able to identify four studies [5, 6, 8, 27] that investigated primary ACL injury, seven that investigated ACL graft and/or CACL ruptures [7, 16, 19, 26, 28–30] and one study [31] that investigated both primary and subsequent ACL injuries. Table 2 summarises key characteristics for studies included in the primary ACL injury meta-analysis and Table 3 contains the characteristics for those included in the subsequent ACL graft or CACL ruptures.

### Primary ACL Injury

**Fig. 2:**
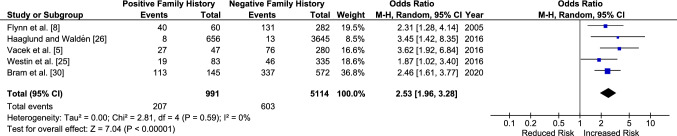
Odds of sustaining a primary anterior cruciate ligament injury with a family history of anterior cruciate ligament injury. The Hägglund and Waldén [27] study includes a female individual-only population. For Flynn et al. [8], only the results from first-degree relatives were used in the meta-analysis. *CI* confidence interval, *M–H* Mantel–Haenszel

There were five studies included in the primary ACL injury analysis (Table 2). All studies found that the odds of sustaining a primary ACL injury are increased in those who have a family history of ACL injury. Collectively, the odds are increased by two and a half times with an OR of 2.53 [95% CI 1.96–3.28, *p* < 0.001] (Fig. 2).

For the studies included in the analysis of primary ACL injury, three were case–control designs with matched controls [9, 23, 30] and two were cohort studies [6, 27]. A sensitivity analysis was completed by separating the results of these studies into two subgroups by the two design types. There was minimal change to the overall ORs and CIs between the cohort study designs [6, 27] (OR 2.32 [95% CI 1.30–4.14, *p* < 0.001]) and the case–control study designs [5, 8, 31] (OR 2.64 [95% CI 1.95–3.58, *p* = 0.001]).

**Fig. 3:**
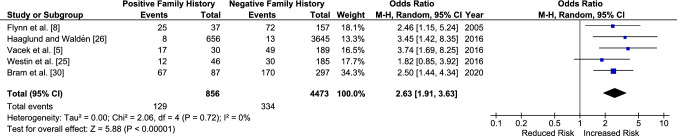
Odds of sustaining a primary anterior cruciate ligament injury in female individuals with a family history compared to those without. *CI* confidence interval, *M–H* Mantel–Haenszel

**Fig. 4:**
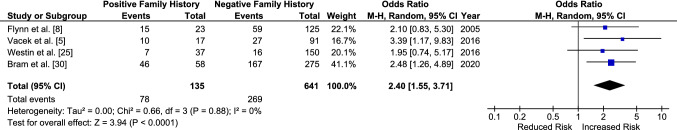
Odds of sustaining a primary anterior cruciate ligament injury in male individuals with a family history compared to those without. *CI* confidence interval, *M–H* Mantel–Haenszel

**Fig. 5:**
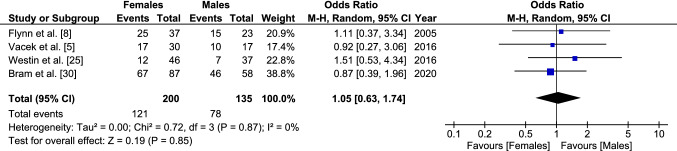
Odds of sustaining a primary anterior cruciate ligament injury in male individuals compared to female individuals in those with a family history. An odds ratio > 1 indicates increased odds for anterior cruciate ligament injury in male individuals. *CI* confidence interval, *M–H* Mantel–Haenszel

The ORs remained similar when the data were separated by sex. Female individuals with a family history of ACL injury were found to have a 2.6 times increased odds of sustaining a primary ACL injury (2.63 [95% CI 1.91–3.63, *p* < 0.001]) (Fig. 3) and male individuals had a 2.4 [95% CI 1.55–3.71, *p* < 0.001] times increased odds (Fig. 4). When compared directly, there was no significant difference in the odds of sustaining an ACL injury between a male and female athlete with a family history of ACL injury (OR 1.05 [95% CI 0.63–1.74, *p* < 0.85]) (Fig. 5).

### Subsequent ACL Injury

**Fig. 6:**
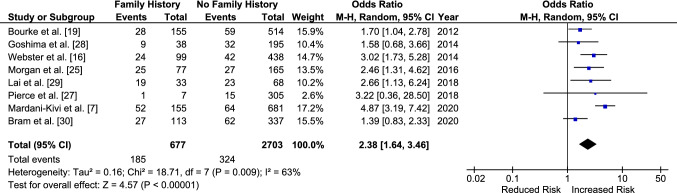
Odds of sustaining a subsequent anterior cruciate ligament injury (anterior cruciate ligament graft rupture or contralateral anterior cruciate ligament injury) in those with a family history compared to those without. Note that for Bourke et al. [19] and Morgan et al. [26], follow-up data at 5 years were used in the meta-analysis. *CI* confidence interval, *M–H* Mantel–Haenszel

There were eight studies included in the analysis of subsequent ACL injury following ACL reconstruction for those with a family history (Table 3). Having a family history significantly increased the odds of sustaining further injury for both ACL graft and CACL injuries compared with those without. Seven studies investigated both ACL graft ruptures and CACL injuries whilst one study, Pierce et al. [28], only investigated CACL injuries. The odds of sustaining a subsequent ACL injury in either knee are 2.38 [95% CI 1.64–3.46, *p* < 0.001] times increased for those with a family history compared with those without (Fig. 6).

**Fig. 7:**
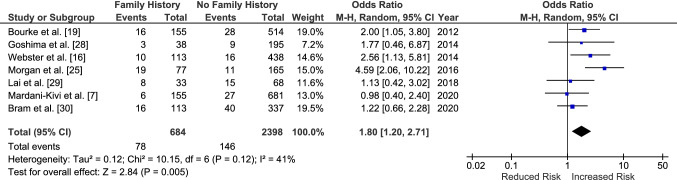
Odds of sustaining an anterior cruciate ligament graft rupture in those with a family history compared to those without. *CI* confidence interval, *M–H* Mantel–Haenszel

**Fig. 8:**
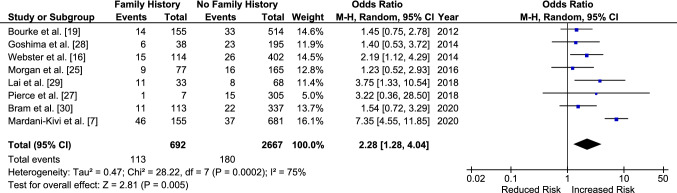
Odds of sustaining a contralateral anterior cruciate ligament injury in those with a family history compared to those without. *CI* confidence interval, *M–H* Mantel–Haenszel

**Fig. 9:**
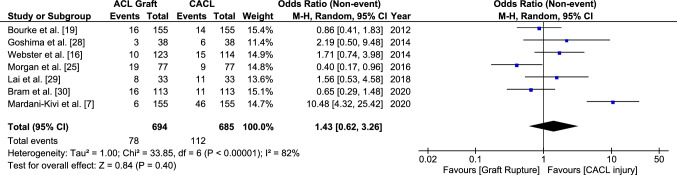
Odds of sustaining an anterior cruciate ligament (ACL) graft rupture compared to a contralateral ACL (CACL) injury in those with a family history. *CI* confidence interval, *M–H* Mantel–Haenszel

Having a family history of ACL injury increased the odds of sustaining a graft rupture with an OR of 1.80 [95% CI 1.20–2.71, *p* = 0.005] (Fig. 7). The odds of sustaining a CACL injury were also increased by 2.28 [95% CI 1.28–4.04, *p* = 0.005] for those with a family history (Fig. 8). There was no significant difference when the ORs for CACL injuries were compared directly to those for graft ruptures (OR 1.43 [95% CI 0.62–3.26, *p* = 0.40]) (Fig. 9).

All studies included in the analysis for subsequent ACL injuries showed moderate-to-high heterogeneity [32]. A sensitivity analysis was completed by individually removing and adding each study to the analysis. Excluding Mardani-Kavi et al. [7] from the analysis reduced all studies to an *I*^2^ of 0% across all outcomes except the odds of sustaining a CACL injury compared to a graft rupture (Fig. 9) in those with a family history, which were reduced from high (82%) to low (41%) heterogeneity [32]. Although the methodological quality in the study by Mardani-Kavi et al. [7] was rated as good, the only difference between this study and the others was that it investigated family history in “siblings only” rather than “1st degree relatives”. This study reported a high OR and small CIs. If this study is removed from the analysis, then the odds of sustaining a subsequent ACL graft rupture or CACL injury reduce to 1.99 [95% CI 1.56–2.55, *p* < 0.001], ACL graft rupture to 1.65 [95% CI 1.16–2.35, *p* = 0.020] and CACL injury to 1.75 [95% CI 1.27–2.40, *p* < 0.001]. A significant effect across all outcomes is still observed even when this study is excluded from the meta-analysis.

## Discussion

This systematic review with meta-analysis focused on the risk for ACL injuries in individuals with a family history of ACL injury. It found that, in athletic populations, those with a family history of ACL injury have an approximately 2.5 times increased odds of sustaining both a primary and subsequent ACL injury than those without a family history. This confirms the significant impact of family history as a risk factor in primary ACL injuries, as well as ACL graft and CACL ruptures.

This review thoroughly investigated family history as a risk factor in relation to primary ACL injuries, graft ruptures and CACL injuries together. A recently published systematic review and meta-analysis of risk factors in CACL injuries by Cronström et al. [33] also found those with a family history were at increased risk and reported an OR of 2.07 (95% CI 1.54–2.80, *p* < 0.001). However, three studies [15, 24, 25] out of the nine used in their analysis were a part of one cohort [19] and the same participants were therefore included multiple times. Our results showed that the impact of family history on CACL injury (OR 2.28) was slightly greater than that reported by Cronström et al. [33]. We are not aware of any other review that has combined data on the risk of family history for a primary ACL injury or graft rupture with which to compare the current results.

It is not clear why family history is a significant risk factor for both primary and subsequent ACL injury. There has been a significant amount of work investigating potential intrinsic factors for ACL injury such as joint hypermobility, tibial slope angle, femoral notch width, ligamentous thickness as well as hormonal and genetic factors [3, 4, 34, 35]. However, there are few studies that investigate the relationship of these intrinsic factors and family history of ACL injury together. Therefore, it is unknown if the risk factor of family history is due directly to these intrinsic factors that have been identified in the research. One study by Keays et al. [36] investigated femoral notch width in ACL-injured siblings compared to non-injured athletic sibling pairs. They found that 50% of the ACL-injured sibling pairs had a narrow femoral notch width compared to none (0%) in the non-injured pairs. Goshima et al. [29] investigated an ACL-injured cohort and compared those with a family history of ACL injury to those without. They found that there were no significant differences in age, height, weight, body mass index and generalised joint laxity between the two groups. However, their results showed that the tibial slope angle was significantly higher in the group of ACL-injured participants with a family history. Increased tibial slope and a narrow femoral notch are two factors that may contribute to the impact of family history in ACL injury [29, 36].

There is also the consideration of extrinsic factors and the impact that this has on family history as a risk factor. It has been found that playing sport increases an individual’s risk of sustaining an ACL injury [37]. Children and adolescents are more likely to participate in sport when their parents and siblings play sport [38]. Goshima et al. [29] investigated the mechanisms of injury in those with a family history of ACL injury. They found that 65% of participants with a family history of ACL injury sustained their primary ACL rupture whilst playing the same sport as their immediate family member. Therefore, a family history of ACL injury may not only be a hereditary factor but also may reflect the individual being part of an active family that participates in potentially higher risk sports. This systematic review with meta-analysis shows that whatever the causes for this familial link, it is a significant risk factor in ACL injury.

Identifying strong relationships between screening test results and injury risk is the first step in potentially reducing injuries [35, 39]. This systematic review has found a significant relationship between family history of ACL injury and increased odds of injury in athletic populations. Screening for family history as a risk factor is easily achievable as it can be assessed by anyone at all levels of sports participation, unlike many of the other intrinsic risk factors. Those who report a family history of ACL injury should be educated to complete injury reduction programmes that have been found to reduce the risk of ACL injury in athletes [18, 40]. Those who then require an ACL reconstruction and want to return to sport should also be encouraged to complete injury reduction programmes post-reconstruction given that the odds of sustaining a graft rupture or CACL injury are double compared with those without. The benefits of an injury reduction programme have not been investigated in a cohort with a known family history of ACL injury and is a potential topic for future research.

There are a number of limitations to this study. First, we were only able to analyse by sex for primary ACL injuries. There was no study that published their data of family history by sex except for Hägglund and Waldén [27] who had a female individual-only cohort and Lai et al. [30] who had a male individual-only cohort. All other sex-based data in this systematic review were obtained by contacting the authors for unpublished data. Most of the studies in the primary ACL injury analysis focused specifically on family history as a risk factor and when contacted had this information by sex available. The studies in the subsequent ACL injury analysis mostly investigated the recurrence of injury and family history was one of several outcomes investigated. We felt that asking the remaining authors of the studies to review their data by sex was beyond the scope of intent of the original papers and would have required most authors to re-analyse their data. As a result, we did not investigate the impact of sex in subsequent ACL injuries for those with a family history. It would be recommended for future studies investigating risk factors for ACL injuries to report their outcomes by sex to improve our understanding of the factors that may contribute to the difference in injury rates between male and female individuals [11].

Second, within the 12 studies included in this analysis, there were inconsistencies regarding the definition of family history. Eight studies [13, 16, 22, 24, 27–30] collected data for first-degree relatives, whilst two studies [5, 6] used data for parents only and one study [7] investigated siblings only. Flynn et al. [8] was the only study to investigate the differences between first-degree, second-degree, and third-degree relatives. They found the odds of sustaining a primary ACL injury increased when the analysis was performed for first-degree relatives (OR 2.24) compared to first-degree, second-degree, and third-degree relatives combined (OR 2.0). Participants are also likely to provide more reliable information regarding first-degree relatives than extended family members. To be able to draw consistent conclusions, the definition of family history should be defined consistently in future research. Given that most of the studies define or investigate family history as first-degree relatives, it would be advised to use this definition.

## Conclusions

This systematic review with meta-analysis provides a comprehensive analysis of ACL injury risk for athletes with a family history of ACL injury compared to those without. Whilst there are only 12 studies, they all have generally high methodologies. For both primary and subsequent ACL injuries, this study has been able to investigate sub-groups for further analysis to provide greater insight into the contribution of this important risk factor.

Having a family history of ACL injury increases the odds of sustaining a primary ACL injury compared to those without a family history by 2.5 times and increases the odds of a subsequent ACL injury by 2.4 times. However, if a family history of ACL injury is present, the sex of the athlete does not increase the risk for primary injury nor is there a difference in the risk for a subsequent graft rupture compared to a CACL injury.

## Declarations

### Availability of data and material

The authors confirm that all relevant data are included in the article.
